# Supplementary material for: Learning curve and surgical outcome of robotic assisted colorectal surgery with ERAS program
Source: Sci Rep. 2022 Nov 29;12:20566. doi: 10.1038/s41598-022-24665-w (PMC9709162; doi:10.1038/s41598-022-24665-w)
Supplement: Supplementary file 1 — Supplementary Information 1. [file 41598_2022_24665_MOESM1_ESM.zip › LSC Raw data-Ñ[▒K/102/H_ñw╝╨░O▒Kñσ.pdf]

正式摘要(電子簽章：00000203050796)

W52 / W52 ☐ 英文標題(注意:無英文姓名-請補病人基本檔!) ☐ 【讀取】憑證

臺中榮民總醫院

出院病歷摘要

轉入號 : 轉出號 :

[redacted]

**(8)**流水編號	
**(9)**入院日期	109/11/04 (2020) 直腸外科 W52 - 112
**(10)**轉科日期	
**(11)**出院日期	109/11/12 (2020) 住院天數計 8 日

診斷

- 入院 :1. S-Colon adenocarcinoma, FAV 30cm, cT3N1M0, Stage IIIB.
  2. SCC of esophagus, TI(30-36cm), cT2N0M0, stage II.
  3. old smoker, suspected COPD lung
- 出院 :1. S-Colon adenocarcinoma, FAV 30cm, cT3N1M0, Stage IIIB, s/p laparoscopic anterior resection on 20201105 2. SCC of esophagus, TI(30-36cm), cT2N0M0, stage II, under neoadjuvant CCRT 3. old smoker, suspected COPD lung

主訴

- Bloody stool for 2 months

病史

- This is a 75 y/o man with history of
  1. SCC of esophagus, TI(30-36cm), cT2N0M0, stage II.
  2. S-Colon adenocarcinoma, FAV 30cm, cT3N1M0, Stage IIIB.
  , who presented with bloody stool for 2 months. UGI and LGI scopy were done later, which iopsy confirmed esophagus and S-colon malignancy. Initially, combined surgery was suggested, but patient refused operation during CS admission (2020/10/21~23) and favored CCRT for esophageal malignancy. This time, he is admitted for operation.

體檢發現

- :
  - General appearance :
    - easy looking , conscious clear , without cardiopulmonary distress
    - Vital signs自動帶入date 體溫(BT) 脈搏(P) 呼吸(R) 血壓(BP) 身高(BH) 體重(BW) BMI
      20201104 36℃ 83次/分 18次/分 144/89mmHg 161.3cm 73.2kg 28.1
  - HEENT :
    - Eyes: no anemic conjunctiva, no icteric sclera, normal EOM
    - ENT: no otorrhea, no rhinorrhea, no erythematous mucosa, no injected tonsil, no deformity
  - Neck: supple, freely movable, trachea midline,no deformity, no engorged jugular vein , no palpable thyroid or lymph node enlargement
  - Chest:
    - Inspection: Grossly normal, symmetric and fully expansion, no accessory muscle used, no operation scar, no spider angiomata, no gynecomastia
    - Palpation: no palpable mass or nodule, no subcutaneous emphysema, no tenderness
    - Ausculation: clear breathing sound
  - Heart:
    - Percussion: normal heart size
    - Ausculation: regular heart beat, normal S1, S2, no gallop, no murmur
  - Abdomen:
    - Inspection: in

-
    - ▪ flat contour, symetrically, no scar, no stria, no dilated veins, no rashes
    - Ausculation: normoactive bowel sound, no abdominal bruits
    - palpation: soft, no muscle guarding or rigidity, no local tenderness, no rebounding pain, no palpable mass, no palpable pulsation
    - Percussion: no shifting dulllness, no hepatomegaly, no splenomagaly
-

Peripheral pulsation :	CA	BA	RA	FA	PA	PTA	DPA
Right side :	++	++	++	++	++	++	++/+++
Left side :	++	++	++	++	++	++	++/+++

- Genito-anus: no tenderness or palpable mass lesion or rectal shelf, normal external genitalia, normal anal tone, brown stool

CRS CONDITION

-
    - Lung

手術日期及方法

- 手術日期及方法20201105 1. Intraoperative colonoscopy : (-)
  2. High ligation (+) : At IMA root. IMV and left colic artery were all resected and ligated via hemo-clips.
  3. The colon was not taken down near the splenic flexure
  4. EndoGIA (60-4.1) X2 + EEA (29mm) were used
  5. Proximal cut end : 10 cm, distal cut end : 3 cm
  6. No drainage tube was inserted
  7. The ADHESION BARRIER FILM was placed below the central umbilicus extended wound.

住院治療經過

- After admission, operation was done on 11/5. However, due to underlying of COPD lung, with post operative atelectasis(found on f/u CXR), the patient need O2 supplement with allpurpose. After self-coughing and lung rehabilitation, the O2 supplement was tappered to nasal cannula. There was no target pathogen from sputum, but for prophylactic use, antibiotic use with Flomoxef and Tapymycin were prescribed since 11/7 and after then. Under relative stable condition, he tried liquid diet since 11/9. Try soft diet on 11/11. MBD was permitted and OPD f/u is scheduled under stable condition.

合併症

一般檢查

特殊檢查

病理報告

其它

出院時情況

- 15.計劃性出院

出院指示

藥名	劑量	劑量單位	用法	頻次	數量	數量單位	現況	天數	單位
OCAS Harnalidge 0.4 mg	1	TAB	PO	QDAC	6	TAB	TH	6	天
Colchicine tab 0.5mg	1	TAB	PO	BID	12	TAB	TH	6	天

出院計劃

主治醫師_2062F 蔣鋒帆 / 住院醫師_2310K 陳瓘夫

正式摘要(電子簽章：**000000203050796**)

W52 / W52

doctorRank:B
